# Supplementary material for: Cooperation is Fleeting in the World of Transposable Elements
Source: PLoS Comput Biol. 2006 Dec 1;2(12):e162. doi: 10.1371/journal.pcbi.0020162 (PMC1676026; doi:10.1371/journal.pcbi.0020162)
Supplement: Supplementary file 1 [file pcbi.0020162.sd001.doc]

Cooperation is Fleeting in the World
 of Transposable Elements
Andreas Wagner
Department of Biology, University of New Mexico, Albuquerque, New Mexico, United States of America
Cooperation in Transposable Elements
Editor: Carl Bergstrom, University of Washington, United States of America
Received April 24, 2006; Accepted October 13, 2006; Published December 1, 2006
Copyright: © 2006 Andreas Wagner. This is an open-access article distributed under the terms of the Creative Commons Attribution License, which permits unrestricted use, distribution, and reproduction in any medium, provided the original author and source are credited.
Abbreviations: ESS, evolutionarily stable strategy; IS, insertion sequence 
* To whom correspondence should be addressed. E-mail: wagnera@unm.edu
Composite transposons are key vehicles for the worldwide spreading of genes that allow bacteria to survive toxic compounds. Composite transposons consist of two smaller transposable elements called insertion sequences (ISs), which flank the genes that permit such survival. Each IS in a composite transposon can either transpose alone, selfishly, or it can transpose cooperatively, jointly with the other IS. Cooperative transposition can enhance an IS's chance of survival, but it also carries the risk of transposon destruction. I use game theory to show that the conditions under which cooperative transposition is an evolutionarily stable strategy (ESS) are not biologically realistic. I then analyze the distribution of thousands of ISs in more than 200 bacterial genomes to test the following prediction of the game-theoretical model: if cooperative transposition was an ESS, then the closely spaced ISs that characterize composite transposons should be more abundant in genomes than expected by chance. The data show that this is not the case. Cooperativity can only be maintained in a transitional, far-from-equilibrium state shortly after a selection pressure first arises. This is the case in the spreading of antibiotic resistance, where we are witnessing a fleeting moment in evolution, a moment in which cooperation among selfish DNA molecules has provided a means of survival. Because such cooperation does not pay in the long run, the vehicles of such survival will eventually disappear again. My analysis demonstrates that game theory can help explain behavioral strategies even for mobile DNA.
Citation: Wagner A (2006) Cooperation is fleeting in the world of transposable elements. PLoS Comput Biol 2(12): e162. doi:10.1371/journal.pcbi.0020162
Synopsis
Molecules can display cooperation and selfish behavior. Wagner investigates cooperative behavior in mobile DNA molecules called transposable elements, which can “hop” among cells, which aid the worldwide spreading of antibiotic resistance genes. A composite transposon consists of two smaller mobile DNA molecules, M, that flank another gene, G, M-G-M. The molecules M are able to change location alone and selfishly. They are also able to change location jointly and cooperatively, thus taking the gene G with them. Cooperation facilitates the spreading of the gene G and of M itself. Cooperativity is advantageous to M. However, Wagner's paper shows that cooperativity is short-lived and not stable on evolutionary time scales. Cooperativity can only be maintained in a transitional, far-from-equilibrium state shortly after a selection pressure first arises. This is the case in the spreading of antibiotic resistance, where we are witnessing a fleeting moment in evolution, a moment in which cooperation among selfish DNA molecules has provided a means of survival. Because such cooperation does not pay in the long run, the vehicles of such survival will eventually disappear again.
Introduction
Composite transposable elements are key vehicles for the worldwide spreading of genes that allow bacteria to survive toxic compounds created by humans. Prominent examples include, on one hand, genes responsible for antibiotic resistance, perhaps the most serious challenge in the human battle against infectious diseases. On the other hand, they include genes for the degradation of toxic industrial waste, genes crucial for cleaning up polluted environments [1–3]. The global scale and intensity at which bacteria are exposed to these stressors has emerged only in the very recent evolutionary past, essentially since the Industrial Revolution.
The transposons that spread these genes are themselves composites—hence their name—of smaller transposable elements called insertion sequences (ISs, Figure 1A). ISs are arguably the simplest transposable elements. They typically consist of one or more genes encoding a transposase necessary for transposition, flanked by two short (10–40 bp) inverted repeats. Between two ISs lie the genes of interest here: their functions are unrelated to transposition, but their presence may permit survival. I will call these genes selectable genes.
Transposition involving a composite transposon or its parts can occur in two ways. First, the two ISs may transpose jointly and thus carry the selectable genes to a different location or a different DNA molecule. I will refer to such transposition as cooperative transposition, because it requires both IS elements. Each of the composite transposon's IS has an outside end (the repeat unit farthest from the selectable genes) and an inside end (the repeat unit nearest to the selectable genes, Figure 1). Cooperative transposition of the entire transposon uses the outside ends of both ISs (Figure 1). Second, any one IS (IS_L_ or IS_R_) may transpose independently from the other. I will call such transposition selfish transposition. Transposition of either IS_R_ or IS_L_ uses the outside and inside ends of the transposed IS (Figure 1).
The key question I will address here is whether cooperative transposition can be an evolutionarily successful strategy from the perspective of an IS—a prototypical example of selfish DNA. One could use several theoretical frameworks to address this question. One such framework is that of population genetics, which could be used to predict the frequencies of ISs and composite transposons within and among genomes. The disadvantage of this framework is its complexity: population genetic models of transposable element dynamics are unwieldy even to study the intragenome evolutionary dynamics [4]. They would get hopelessly complicated for the intergenome dynamics that need to be analyzed for this purpose.
Another candidate framework is game theory. Originally devised to understand conflicts among humans, institutions, and governments [5], it can serve to decipher many aspects of nonhuman behavior [6]. In recent years, game theory has been applied to increasingly small-scale biological phenomena. For instance, it can help explain the diversity of microbial communities, the emergence of cooperativity in bacterial populations, cross-feeding behavior in different bacterial strains, and the evolution of selfishness in bacteriophages [7–12]. Game theory predicts successful behavioral strategies, which in nonhuman organisms are maintained through natural selection.
Game theory has several advantages for my purpose. First, at its core is the concept of the ESS. Whether selfishness, cooperativity, or a mix thereof is an ESS can be easily determined by focusing on the individual composite transposon and its evolutionary fate, without the unnecessary detour of calculating all the quantities important to population genetics. Second, game theory very naturally captures the fact that a composite transposon consists of two “actors”—the ISs—that can behave independently of each other, but whose interaction is critical for the fate of the whole. Game theory is powerful precisely in situations where such interactions matter, whereas population genetics needs to capture interactions through more complex concepts such as frequency-dependent selection [13]. Third, and relatedly, game theory very naturally accommodates the fact that IS_L_ and IS_R_ can have different structures and thus different “interests” in propagating the whole.
Finally, I note a peculiar feature of the evolutionary dynamics of composite transposons. Selfish and cooperative transposition have different effects on an IS's reproductive success only if the transposition product enters a piece of DNA horizontally transferred to a new host (such as a conjugative plasmid or a prophage). In this case, cooperative transposition will allow the new host to survive if it is exposed to an environment requiring the selectable genes. From the perspective of one of the ISs, cooperative transposition then allows the IS to gain one copy of itself (in a new genome). In the case of selfish transposition, a horizontally transferred transposition product (IS_L_ or IS_R_) may perish with its new host in an environment where the selectable genes are needed. Game theory very naturally accommodates this feature, because it conceives of two ISs in a composite transposon as two agents that maximize a payoff. The payoff currency is the likelihood that a new host organism (of the same or of a different bacterial “species”) gains a copy of one of the ISs.
As a corollary of these observations, the intragenome dynamics of transposable elements is irrelevant for the evolutionary fate of cooperative transposition, because the key difference between the reproductive success of selfish and cooperative transposition comes from the dynamics of transposable elements between genomes. This is another reason to eschew more complex population genetic models.
Cooperativity is by no means an assured ESS for transposable elements, because it carries a cost. Aside from successful transposition, transposase activity can trigger DNA rearrangements with a variety of outcomes. These include deletion of the selectable genes, of DNA adjacent to the transposon, or of either of the two ISs. In addition, two or more IS copies in a genome can serve as recombination targets to the host's homologous DNA recombination machinery, which may lead to various DNA rearrangements independent of transposition. Finally, spontaneous excision (and loss) of the composite or of either IS can also occur. Rates of rearrangement or loss, both as a byproduct of transposition and through the host's DNA recombination machinery, are higher for composite transposons than for isolated ISs. The reason lies in the greater number of repeat units in composites. They can simply align inappropriately in more ways than the two inverted repeats of a single IS. [14–18].
Game-theoretic studies have two key prerequisites. First, genetic variation in the behaviors under question must exist; otherwise natural selection could not possibly reach any ESS. It is thus crucial to note that the propensity of an IS to transpose selfishly is determined by its structure. Most important in this regard are the ends and their interaction with the transposase. Multiple mutations in IS ends exist that affect transposition activity of either IS element or the composite. In some composite transposons such as Tn5, transposase may also preferentially act on outside rather than inside ends, thus leading to an elevated rate of cooperative transposition [1,15,19,20]. A second preprequisite is that the success of an agent's action should depend on the other agent's action. This prerequisite is also clearly met: a player (IS) with a propensity to transpose cooperatively (or selfishly) has a payoff that depends on the other player's strategy, embodied in its DNA sequence.
The approach I take examines the conditions under which selfishness, cooperation, or a mix thereof are ESSs for an IS. Natural selection will lead to the accumulation of transposable elements in bacterial genomes whose DNA embodies such an ESS. If selfish transposition is the only ESS, composite transposons cannot persist in the long run. If pure cooperation is the only ESS, composite transposons that do not allow selfish transposition (e.g., because of inactive inside ends) arise and accumulate in prokaryotic genomes. My results suggest that the conditions favoring composite transposition are rarely met, if ever. I provide corroborating evidence by analyzing the distribution of IS spacing in 28 different IS families occurring in more than 200 completely sequenced bacterial genomes.
Results
The Model
An IS's payoff is the expected increase in the number of host genomes that harbor a copy of it. Three factors can affect this payoff's value. They are represented by three model parameters. The first, t (“transposition/transfer”), is the (joint) probability that transposition occurs and that the transposition product undergoes horizontal transfer to a new host genome. Of course, horizontal gene transfer can also occur without transposition. If so, the payoff is independent of transposition, so I need not consider it. Conversely, transposition can occur without horizontal transfer, but the reproductive success of an IS is identical for selfish and cooperative transposition within a genome and thus also need not be considered further. The second parameter, s (“selection”), is the probability that the host cells are subject to an environment in which the selectable genes are necessary for survival. The old host cell will survive because the original host typically retains a copy of the transposable element [3]. However, the new host will survive (and the IS's payoff will be positive) only if the ISs acted cooperatively. Third, there is r (“rearrangement”), the probability of a failed transposition event that leads to a DNA rearrangement eliminating either the IS or perhaps even killing the cell.
Payoffs for IS_L_ and IS_R_
To begin with, I will focus on a scenario in which the payoff is identical for both  IS_L_ and IS_R_ of a composite.
Let us first consider the case, “SS,” where the composite has a structure that favors selfish transposition of both ISs. (For example, both inner ends may be fully functional, or the ISs may be far apart, thus disfavoring cooperative transposition, because transposases act preferably in cis [21].) What is the payoff to one IS, e.g., IS_L_ in this case? With probability ½, it is IS_L_ that undergoes the transposition event, and with probability ½ it is IS_R_. In the first case, the payoff to IS_L_ can be written as P_SS_ = (1 – t) × 0 + t[(1 – s) × 1 + s × 0] = t(1 – s), because the transposable element will gain an additional copy only if it is transposed and transferred to another cell (probability t), where that cell is not under selection for the presence of the selectable genes (probability 1 – s). In the second case, where IS_R_ is transposed, the payoff to IS_L_ is zero. On average, the payoff to IS_L_ is thus
P_SS_ = ½ t(1 – s)         (1a)
Next, consider the case “CC,” where the transposon's structure strongly favors cooperative action. In this case, the payoff to IS_L_ (as well as to IS_R_) calculates analogously as (1 – t) × 0 + t[(1 – s) × 1 + s × 1] = t.
So far, I have not considered the risk of transposon loss due to a DNA rearrangement caused by transposition. Such rearrangements occur preferentially for cooperative transposition rather than for selfish transposition. For example, in the transposition of the transposon Tn10, such arrangements occur at a rate of 10^−5^ per cell and generation, whereas for its constituent IS, IS10, this rate is on the order of 10^−8^ per generation [15]. Preferential loss of ISs during cooperative transposition will lead to a payoff of (−1) with probability r. In sum, one gets
P_CC_ = (1 – r)t – r = t – r(1 + t)	(1b)
The next step consists of the calculation of P_CS_, the payoff to IS_L_ if IS_L_ acts cooperatively whereas IS_R_ acts selfishly. Biologically, this scenario corresponds to a composite in which IS_L_ has undergone a mutation, such that it cannot use its own inside end, but only the outside end of IS_R_ for transposition, which is thus cooperative. IS_R_ on the other hand can use its own inside end. The overall outcome in this case is either selfish transposition of IS_R_ (P_CS_ = 0) or cooperative transposition (P_CS_ = t – r(1 + t)). Which of these outcomes occurs may depend on whether a transposase expressed from IS_L_ or IS_R_ initiates the transposition event, which may be a stochastic event. To allow all possible outcomes and keep the model general, I will thus introduce another parameter, ä (for “dominance”) that ranges from 0 to 1 and indicates the propensity of either outcome. If ä = 1, then P_CS_ = 0. In this case, selfishness is dominant over cooperativity, in the genetic sense, not in the game-theoretic sense [22]. If ä = 0, then P_CS_ = t – r, in which case cooperativity is dominant over selfishness. For 0 < ä < 1, the payoff is intermediate, that is,
P_CS_ = (1 – ä)[t – r(1 + t)]	(1c)
The same reasoning applies to the calculation of P_SC_, the payoff to IS_L_ if IS_R_ acts cooperatively whereas IS_L_ acts selfishly. If ä = 0, then P_SC_  = t – r(1 + t). If ä = 1, then P_SC_ = t(1 – s). (The factor ½ entering P_SS_ does not occur here, because selfish transposition of IS_R_ is not an option.) For 0 < ä < 1, we get
P_SC_ = [t – r(t + 1)] + ä[r(t + 1) – ts]	(1d)
In sum, one obtains the payoff matrix p of Figure 1B, which shows the payoff to IS_L_ in standard game-theoretic notation, with the approximation r(t + 1) ≈ r (valid because t << 1) . I analyze the game for the full matrix as defined by Equation 1 in Protocol S1), but will use this approximation to discuss the results here.
IS_L_ and IS_R_ May Not Receive the Same Payoffs
So far, I have tacitly assumed that IS_L_ and IS_R_ have the same expected payoffs in the same kind of transposition event. This, however, need not be the case. Aside from variation in their ends, IS_L_ and IS_R_ may also encode transposases with slightly different target specificities. This is not far-fetched. For example, different members of the IS3 family generate target site duplications of different lengths and also show different target specificities [23]. The same holds for members of the IS5 family, where one subfamily (ISL2) shows a target preference for the nucleotide triplet ANT, another (IS1031) shows a preference for TNA, and yet others (ISH1, IS903) show no marked target specificities [23]. As a result, IS_L_ and IS_R_ may have different likelihoods t of being transposed into a mobilizable plasmid or prophage and thus be transferred to a new host. Analogous variation may exist for the ISs' propensity to undergo DNA rearrangement after transposition. Such differences naturally lead to the notion of an asymmetric game, which is characterized by two payoff matrices. Their structure is analogous to that of p from Figure 1B and is shown in Equation 7 of Protocol S1.
Cooperativity Is an ESS Only if DNA Rearrangements Are Much Rarer than Horizontal Transfer
I will now briefly summarize the most important results of the model, whose behavior is exhaustively analyzed in Protocol S1. If both ISs are very similar and thus receive the same payoff, selfish transposition is the only ESS as long as
		(2a)
Cooperative transposition is an ESS, either alone or in conjunction with selfish transposition as a second ESS, only if
r < st		(2b)
Notice that s < (1 + s)/2 < 1.
The relationship of Equation 2 is very simple and holds for all values of ä, that is, regardless of whether selfishness is genetically dominant (ä = 1), recessive (ä = 0), or intermediate. The only region of parameter space not covered by Equation 2 is the narrow region where st < r < t(1 + s)/2. Here  the value of ä matters. If selfishness is dominant (ä = 1)—the most favorable case for selfish transposition—then selfish transposition is already an ESS if r > ts, otherwise cooperative and selfish transposition coexist as stable ESSs (Figure 1C; and Figure S2 in Protocol S1). Selfish dominance is biologically most realistic: envision a composite transposon whose IS_L_ can transpose selfishly, but whose IS_R_ transposes cooperatively. (It may have a mutated inside end and preferably transpose using the outside end of IS_L_.) Because transposases act preferentially in cis [21], and because the likelihood of successful transposition decreases dramatically with the distance of the IS ends, a successful (cooperative) transposition event initiated from IS_R_ is much less likely than a selfish transposition event initiated from IS_L_. However, even if cooperativity were dominant (ä = 0), results would not change dramatically. In this case selfish transposition is an ESS if r > t(1 + s)/2, otherwise cooperativity is the only ESS. A mixed ESS exists only in a very narrow interval of the parameter range (Figure S2 in Protocol S1) and is thus unlikely to be of much biological relevance.
For the realistic case of selfish dominance, cooperativity is never the sole ESS and always coexists with the selfish ESS. In this case, the actually attained ESS depends on the initial condition of the evolutionary dynamics. The ESS will be selfish transposition if the composite, when first established, consists of selfishly transposing ISs. The ESS will be cooperative transposition if both ISs initially transpose cooperatively. Composite transposons most likely originate by the transposition of an IS into its own neighborhood on a DNA molecule, such that original and copy come to be separated by one or a few (selectable) genes. This means that the initial condition of the evolutionary dynamics is close to the all-selfish state, unless a mutation in one of the ISs occurs as a result of this initial transposition. In consequence, selfish transposition is the evolutionarily stable outcome also in this case. Cooperative transposition has a chance to emerge only if r << st, because then the basin of attraction of the selfish ESS is very small.
Despite its much greater mathematical complexity, the asymmetric game, where the two ISs derive different benefits from selfish versus cooperative transposition, yields very similar results. In Protocol S1, I analyze the asymmetric model, as well as a complementary, nongame theoretic analysis, both of which have similar results.
In sum, cooperative transposition can emerge as an ESS only if the risk r of DNA rearrangements as a result of transposition is much smaller than the product of the likelihood t of transposition/horizontal transfer of the transposable element, and the probability s that the new host survives only if it acquires the selectable genes.
Prokaryotic Genomes and Plasmids Are Not Enriched for Composite Transposons
If cooperative transposition was a frequently attained ESS, prokaryotic genomes should contain (stable) composite transposons in abundance. I tested whether this is the case. In genomic DNA, candidate composite transposons are easily identified as (i) two closely linked ISs (e.g., through intact transposase open reading frames) that are (ii) separated by a small number of genes. Both criteria are necessary (although not sufficient) for a functional composite. I assessed the abundance of such candidate composites for members of 28 different IS families in 376 different completely sequenced bacterial genomes and plasmids (202 complete genomes).
The first notable observation regards the distribution of the distance between nearest-neighbor ISs, the two ISs that are most closely spaced among all ISs of a given family in an examined genome. Among a total of 5,901 nearest-neighbor IS pairs I examined here, only the tiny fraction of 3.9% (233) were spaced less than 30 kbp apart. I included only these pairs of ISs in subsequent analyses, because virtually all known composite transposons are less than 30 kbp long [1]. Next, I asked for each genome or plasmid, and for each IS family, separately, whether the number of adjacent ISs with a distance between 0.5 and 30 kbp is greater than expected by chance alone, given the total number of ISs in the genome or plasmid. I did so by employing a randomization assay that reshuffles the existing ISs within a DNA molecule (see Materials and Methods). The answer is shown in Figure 2A. Only one genome contains more IS pairs (three) in the above distance bracket than expected by chance alone (Bonferroni-corrected p < 0.01). The genome is that of Yersinia pestis strain CO92, and the IS is IS21.
In a second, more fine-grained approach, I asked whether the distance between the closest ISs of a given kind in a DNA molecule is smaller than expected by chance alone, using the same randomization method. Figure 2B shows the example of IS91 in the genome of Rhodopirellula baltica. In this analysis, the distance of the closest nearest-neighbor pair in the above Yersinia pestis species is not smaller than expected by chance alone (p = 0.6953). Figure 2C shows the results of this analysis for all 28 IS families and for all genomes. Only four IS pairs show a Bonferroni-corrected p < 0.05. In three of them, the ISs are adjacent to each other, with no intervening genes. The fourth involves two IS630 in Yersinia pseudotuberculosis IP 32953 spaced at less than 2 kbp. Repeating this analysis more conservatively, with IS pairs of different maximum distances, yields very similar results. Figure S4 shows an example involving ISs no more than 10 kbp apart. In sum, there is no evidence for an overabundance of closely spaced ISs with intervening genes in the examined genomes and IS families. If anything, the data suggest that such ISs may be underabundant. For example, the many p-values close to one in Figure 2A suggest that genomes contain few closely spaced IS pairs.
Discussion
A scenario creating evolutionarily stable composite transposons with cooperatively acting ISs begins with the random transposition of two identical (selfish) ISs to nearby locations in the genome. If there are genes between these ISs that can confer an advantage to a new host after horizontal transfer, natural selection may favor transposition of the resulting composite. DNA changes that cause preferential cooperative transposition may then occur, and result in the preferential propagation of such (stabilized) composites. Potential examples of such DNA changes are mutations in the transposase genes, in the IS ends, or in both, that lead to preferential use of transposon outside ends (Figure 1A).
Cooperative transposition can emerge as an ESS only if the risk r of transposon rearrangements resulting from transposition is much smaller than the product of the likelihood t of transposition followed by transfer of the transposon, and the probability s that the new host survives only if it acquires the selectable genes. The smaller s is, the weaker the selective advantage of cooperative transposition, and the smaller the chances that these conditions are met. The most favorable condition for the emergence of cooperativity is that of very strong selection (s = 1). In this case, r < t is necessary for the emergence of cooperativity.
Quantitative data on rates of potentially destructive DNA rearrangements are available for the composite transposon IS10. Such rearrangements occur at a rate of approximately 10^−5^ per cell and generation. These events are promoted by the transposase activity of Tn10 (as opposed to by the mere presence of its two repeat sequences). This number becomes striking if one considers that the rate of transposition for this composite transposon is 10^−7^, or two order of magnitudes lower. This means that only one in 100 initiated transposition events may lead to normal transposition [15,18]. DNA rearrangements in the course of the transposition process are the rule rather than the exception.
Chromosomal gene transfer under laboratory conditions via conjugative plasmids such as the F-factor from laboratory strains of Escherichia coli occur typically at a rate of 10^−5^ to 10^−7^ per donor cell, and may be much higher in some strains. Despite an abundance of data on horizontal transfer under laboratory condition, much less is known about horizontal transfer in the wild. Not surprisingly, rates of horizontal transfer are often low in the wild [24]. For example, conjugal transfer frequencies of an IncQ plasmid from E. coli to Ralstonia eutropha in the rhizosphere of wheat occurs at frequencies of 0.5 × 10^−10^ per donor cell [25]. Tetracycline resistance genes are transferred through bacterial conjugation between strains of the bacterium Arcanobacterium pyogenes, a pathogen of domestic animals, at rates between 2.5 × 10^−10^ and 4.4 × 10^−9^ per donor cell [26]. In an example involving not conjugation but viral transduction of the composite transposon Tn5, Jiang and Paul [27] found transduction rates between 5.13 × 10^−9^ and 1.33 × 10^−7^ per colony-forming unit among cells of marine communities. Transduction rates per donor cell would be lower, because not all donor cells may harbor fully infectious phages. In addition, all these rates do not account for the probability that a transposable element first has to transpose into a mobilizable piece of DNA, such as a prophage or a conjugative plasmid. This probability is also small, because such DNA molecules are small transposition targets compared with the bacterial chromosome.
Taken together, these limited data suggest that rates of DNA arrangement associated with transposition are by no means smaller than rates of horizontal transfer, as required for the evolution of stable cooperative transposition. And even if they were smaller, cooperative transposition would face an additional obstacle: especially for dominant selfishness, cooperative and selfish transposition may be coexisting ESSs, and cooperativity can arise only if the ISs of a newly created composite transposon already transpose preferentially jointly. This condition is unlikely to be met, precisely because composite transposons initially arise from selfish transposition of ISs.
In sum, cooperative transposition is not an ESS of ISs. By implication, higher-order composites (composite transposons made of composite transposons) would also not be evolutionarily stable.
Every mathematical model abstracts from immensely complex natural phenomena by neglecting some factors that may be of limited importance. One such factor is the evolutionary dynamics of transposable elements within a genome. The prime reason is that the adaptive differences between selfish and cooperative transposition only emerge if one considers the dynamics among genomes.
Another neglected factor is that a transposition event can lead to the elimination of the transposed IS at its original location [1]. As opposed to the DNA rearrangements that the model does represent, such nonreplicative transposition does not destroy a transposable element but leaves only one instead of two copies of the element in the genome. Nonreplicative transposition affects mostly the dynamics of transposable elements within a genome, but the key difference between selfish and cooperative transposition emerges from the dynamics among genomes. (Horizontal transfer typically leaves a transposable element in the original genome). In addition, nonreplicative transposition probably affects individual ISs and composite transposons in similar ways, because it results from the mechanics of the transposition process itself.
A third factor not modeled here is that the ISs of composite transposons are two closely spaced, highly similar DNA repeats, which are good targets for a cell's recombination machinery. Pertinent data come from the rate of precise excision of transposable elements, which may not be triggered by transposition itself. The result of such excision is a complete loss of a transposon from a genome. The excision rate has been estimated at 10^−6^ for the composite Tn5. In contrast, the excision rate for IS50, the constituent IS of Tn5 is 3 × 10^−9^ [16]. Similarly, the excision rate for Tn 10 is 10^−9^, and for Tn10's constituent IS, IS10, it is an order of magnitude lower [15,18]. Some composite transposons, such as Tn5271 [28] may have loss rates as high as 10^−3^ per cell and generation. The recombinational instability of composite transposons may thus further disfavor cooperative transposition.
Taking these observations together, it is perhaps less surprising that an analysis of more than 200 bacterial genomes finds very few closely linked IS pairs that might constitute composite transposons, and no excess—perhaps even a deficiency—of such pairs from what would be expected by chance alone. A lack of available raw material for natural selection—variation in IS element spacing—is not likely to account for this pattern. Estimates of transposition rates for bacterial ISs and similar transposable elements in eukaryotes range over two orders of magnitude, between 10^−3^ to 10^−5^ per infected cell and generation [1,4,18,29–31]. Importantly, this rate of transposition refers to successful transposition events, events without strongly deleterious effects that would kill the host. In populations like that of E. coli with effective sizes on the order of 10^8^, and a number of 100–300 generations per year in the wild [32,33], this implies on the order of 10^5^ − 3 × 10^7^ transposition events per population and year. Because many ISs can insert into multiple sites not only in a genome but even in a stretch of DNA on the order of a gene's length, many such events may lead to close spacing of ISs, from which natural selection might recruit composite transposons with potentially useful genes. Similarly, the enormous success of horizontal gene transfer in bacterial genome evolution suggests that plenty of genes can prove useful to a new host after their horizontal transfer [3].
Although the data support the model predictions, an apparent conundrum remains: composite transposons do exist and are successful vehicles of gene transfer in situations of extremely high anthropogenic pressure on bacterial populations. The most notable example is the evolution of antibiotic resistance. The following argument shows that this observation is fully consistent with the absence of cooperativity as an ESS: composite transposons may be abundant in far-from-equilibrium situations.
Imagine two bacterial species, 1 and 2, with similar genome composition and IS content. Genome 1 has a gene G necessary for survival in a specific environment, whereas genome 2 lacks this gene, and needs to acquire it via transposon-mediated horizontal transfer. Among the many individual transposition events occurring in population 1 over short evolutionary time scales, some may lead to the emergence of a composite transposon T in which two ISs flank the gene G. If µ is the rate at which T arises and if í is the rate at which T disappears again (through random transposition of ISs), then population genetic theory [34] tells us that a mutational equilibrium will arise in population 1. The frequency of T at equilibrium is p = µ /(µ +  í) << 1. Transposon T is necessary for the transfer of G from population 1 to population 2, but once population 2 has acquired the gene G, T becomes dispensable again. Within genomes of population 2, the ISs flanking G are then free to transpose to new locations. In the long run, some (low) mutational equilibrium frequency p of T, similar to that in population 1, will be attained. This argument can be easily extended to metapopulations of multiple species. In the long run, all populations in such a metapopulation will have acquired G, but the frequency of T in each population may be very low.
In sum, we see that transposable elements can be important for gene transfer without requiring their stable maintenance, as would occur in an evolutionarily stable equilibrium. High frequencies of transposable elements can only be maintained in a transitional, far-from-equilibrium state shortly after a selection pressure first arises. This is exactly the case for antibiotic resistance in clinical isolates of bacterial populations.
We are witnessing a fleeting moment in evolution, a moment in which cooperation among selfish DNA molecules has provided a means of survival. Because such cooperation does not pay in the long run, the vehicles of such survival will eventually disappear again.
Materials and Methods
Analysis of insertion sequence distribution.
The files listed under Accession Numbers comprise the DNA sequence and annotation for 202 completely sequenced bacterial genomes as well as multiple extrachromosomal DNA molecules. The ISs IS1, IS2, IS3, IS51, IS150, IS407, IS4, IS5, IS427, IS903, IS1031, ISH1, ISL2, IS6, IS21, IS30, IS66, IS91, IS110, IS200, IS605, IS256, IS630, IS982, IS1380, ISAs1, and ISL3 are representative members of all 28 major families of bacterial ISs [23]. I determined the position and pairwise distance of 5,967 transposase genes annotated as belonging to these ISs in all of the above chromosomal and extrachromosomal sequences. Sixty-six of the DNA molecules examined contained only one transposable element. The remaining 5,967 − 66 = 5,901 ISs occurred in more than one copy per genome or plasmid. This means that there are 5,901 pairs of ISs of the same family that could potentially form the ISs which constitute a composite transposon. However, most of these pairs are not viable candidates for the IS elements constituting a composite transposon. First, many of them are too far apart. (Most known composite transposons are less than 30 kbp in length.) Second, some of them are too close together to harbor genes between them that are necessary to constitute a composite transposon. Thus I restricted myself in subsequent steps of my analysis to nearest-neighbor ISs of the same IS family whose beginning points (upstream-most position on the Watson strand) were at least d_−_ = 500 bp apart, and that had no more than a maximum distance of d_+_ = 10 – 30 kb (Figure 2; and Figure S4 in Protocol S1).
For a given genome with a given number k of ISs in a given family, I first determined the number of nearest-neighbor IS pairs (“candidate pairs”) that meet these criteria, and the pair with the smallest nearest-neighbor distance. I then asked whether candidate pairs, both collectively and individually, were significantly closer in the genome than expected by chance alone. I used a randomization test to address this question. Specifically, I first determined the positions of the n midpoints of all intergenic regions in the genome. I then distributed the k ISs randomly among the n midpoints, and determined (i) the number of (randomized) IS pairs in the distance interval (d_−_, d_+_), and (ii) the distance in bp between the nearest neighbors. I repeated this process 10,000 times, which yields a distribution of randomized nearest-neighbor distances. I recorded as a p-value the fraction of reshuffling events in which (i) more IS pairs are in the length interval (d_−_, d_+_), and (ii) the pairwise distance of an IS pair was shorter than for the randomized ISs. Small p-values mean that (i) more ISs in IS pairs are close together, and (ii) the ISs in the shortest IS pair are significantly closer than expected by chance alone. I repeated this test for all IS pairs, that is, in all of the 28 families and in all of the examined DNA molecules. The test implicitly assumes that transposition into noncoding regions has a higher likelihood of survival. I note that the test would be more conservative if the reshuffling among noncoding regions was relaxed, for example, by allowing insertions anywhere in noncoding regions, because in this case the randomized ISs could be closer together, which might lead to an increase (lower significance) of the p-values observed. In other words, I would observe even fewer significant p-values.
Supporting Information
Protocol S1.
Supplementary Online Results
Found at doi:10.1371/journal.pcbi.0020162.sd001 (151 KB DOC).
Accession Numbers
I obtained all 376 genbank files that were available for bacterial genomes from the National Center for Biotechnology Information (NCBI) in February 2005 (ftp://ftp.ncbi.nlm.nih.gov/genbank/genomes/Bacteria/): RefSeq ID NC_000117 to NC_007530.
Acknowledgments
I would like to thank Carl Bergstrom and Jim Bull, as well as two anonymous reviewers, for thoughtful comments.
Author contributions. AW conceived and designed the experiments, performed the experiments, analyzed the data, contributed reagents/materials/analysis tools, and wrote the paper.
Funding. Support through US National Institutes of Health grant GM 63882 is gratefully acknowledged.
Competing interests. The author has declared that no competing interests exist.
References
1. Berg DE, Howe MM, editors (1989) Mobile DNA. Washington (D.C.): ASM Press. 972 p.
2. Nojiri H, Shintani M, Omori T (2004) Divergence of mobile genetic elements involved in the distribution of xenobiotic-catabolic capacity. Appl Microbiol Biotechnol 64: 154–174.
3. Bushman F (2002) Lateral DNA transfer: Mechanisms and consequences. Cold Spring Harbor (New York): Cold Spring Harbor University Press. 448 p.
4. Charlesworth B, Langley CH (1989) The population genetics of Drosophila transposable elements. Annu Rev Genet 23: 251–287.
5. von Neumann J, Morgenstern O (1953) Theory of games and economic behavior. Princeton (New Jersey): Princeton University Press. 641 p.
6. Maynard-Smith J (1982) Evolution and the theory of games. Cambridge (United Kingdom): Cambridge University Press. 224 p.
7. Griffin AS, West SA, Buckling A (2004) Cooperation and competition in pathogenic bacteria. Nature 430: 1024–1027.
8. Czaran TL, Hoekstra RF, Pagie L (2002) Chemical warfare between microbes promotes biodiversity. Proc Natl Acad Sci U S A 99: 786–790.
9. Turner PE, Chao L (2003) Escape from prisoner's dilemma in RNA phage phi 6. Am Nat 161: 497–505.
10. Turner PE, Chao L (1999) Prisoner's dilemma in an RNA virus. Nature 398: 441–443.
11. Velicer GJ, Yu YTN (2003) Evolution of novel cooperative swarming in the bacterium Myxococcus xanthus. Nature 425: 75–78.
12. Pfeiffer T, Schuster S, Bonhoeffer S (2001) Cooperation and competition in the evolution of ATP-producing pathways. Science 292: 504–507.
13. Nowak MA, Sigmund K (2004) Evolutionary dynamics of biological games. Science 303: 793–799.
14. Gray Y (2000) It takes two transposons to tango. Trends Genet 16: 461–468.
15. Kleckner N (1989) Transposon Tn10. In: Berg D, Howe M, editors. Mobile DNA. Washington (D.C.): American Society for Microbiology Press. pp. 211–226.
16. Berg D, Egner C, Hirschel B, Howard J, Johnsrud L, et al. (1981) Insertion, excision, and inversion of Tn5. Cold Spring Harb Symp Quant Biol 45: 115–123.
17. Berg D (1989) Transposon Tn5. In: Berg D, Howe M, editors. Mobile DNA. Washington (D.C.): American Society for Microbiology Press. pp. 185–210.
18. Shen MM, Raleigh EA, Kleckner N (1987) Physical analysis of Tn10 and IS10-promoted transpositions and rearrangements. Genetics 116: 359–369.
19. Sasakawa C, Berg DE (1982) IS50-mediated inverse transposition. Discrimination between the two ends of an IS element. J Mol Biol 159: 257–271.
20. Phadnis SH, Berg DE (1987) Identification of base pairs in the outside end of insertion sequence IS50 that are needed for IS50 and Tn5 transposition. Proc Nat Acad Sci U S A 84: 9118–9122.
21. Morisato D, Way JC, Kim HJ, Kleckner N (1983) Tn10 transposase acts preferentially on nearby transposon ends in vivo. Cell 32: 799–807.
22. Osborne M (2004) An introduction to game theory. New York: Oxford University Press. 550 p.
23. Mahillon J, Chandler M (1998) Insertion sequences. Microbiol Mol Biol Rev 62: 725–774.
24. Droge M, Puhler A, Selbitschka W (1999) Horizontal gene transfer among bacteria in terrestrial and aquatic habitats as assessed by microcosm and field studies. Biol Fert Soils 29: 221–245.
25. van Elsas JD, Trevors JT, Starodub ME (1988) Bacterial conjugation between Pseudomonads in the rhizosphere of wheat. FEMS Microbiol Ecol 53: 299–306.
26. Billington SJ, Songer JG, Jost BH (2002) Widespread distribution of a tet W determinant among tetracycline-resistant isolates of the animal pathogen Arcanobacterium pyogenes. Antimicrob Agents Chemother 46: 1281–1287.
27. Jiang SC, Paul JH (1998) Gene transfer by transduction in the marine environment. Appl Environ Microbiol 64: 2780–2787.
28. Nakatsu C, NG J, Singh R, Straus N, Wyndham C (1991) Chlorobenzoate catabolic transposon Tn5271 is a composite class I element with flanking class II insertion sequences. Proc Natl Acad Sci U S A 88: 8312–8316.
29. Hartl DL, Dykhuizen DE, Miller RD, Green J, de Framond J (1983) Transposable element IS50 improves growth rate of E. coli cells without transposition. Cell 35: 503–510.
30. Egner C, Berg DE (1981) Excision of transposon Tn5. Proc Natl Acad Sci U S A 78: 459–463.
31. Kleckner N (1990) Regulating Tn10 and IS10 transposition. Genetics 124: 449–454.
32. Hartl DL, Moriyama EN, Sawyer SA (1994) Selection intensity for codon bias. Genetics 138: 227–234.
33. Ochman H, Elwyn S, Moran NA (1999) Calibrating bacterial evolution. Proc Natl Acad Sci U S A 96: 12638–12643.
34. Hartl DL, Clark AG (1997) Principles of population genetics. Sunderland (Massachusetts): Sinauer. 542 p.
Figure 1. Selfish and Cooperative Transposition, and the Game ISs Play
(A) shows the basic structure of a composite transposon. The narrow open bar indicates the selectable gene(s). The wide bar inscribed with IS_L_ and IS_R_ indicates the ISs flanking these genes. Gray triangles indicate the inverted repeats that constitute the inside and outside ends of the ISs. Red triangles indicate repeat sequences used in a particular type of transposition event. The upper panel illustrates selfish transposition, where either IS transposes independently from the other. The lower panel illustrates cooperative transposition, where the composite transposes as a whole, taking the selectable genes with it.
(B) The structure of the payoff matrix P describing the payoff to IS_L_ in the symmetric game, where both ISs have the same target specificity and the same likelihood of undergoing DNA rearrangement. The parameter t is the joint probability of transposition and horizontal transfer of the transposition product, r is the probability of DNA rearrangement associated with cooperative transposition, and s is the probability that the new host of a transposable element will need the selectable genes for its survival. Entry P_SC_ is the payoff to IS_L_ if a selfish IS_L_ is paired with a cooperative IS_R_ in a composite, and analogously for the other entries of P. The payoff matrix for IS_R_ is the transpose of P.
(C) Schematic diagram of the evolutionary stable strategies (ESSs) in the case of genetic dominance of selfish transposition. Selfish transposition (“S”) is the only ESS if r > ts. Selfish and cooperative transposition (“C”) are both ESSs if r < ts.
doi:10.1371/journal.pcbi.0020162.g001
Figure 2. No Significant Enrichment of Composite Transposons in 376 Completely Sequenced Bacterial Genomes and Plasmids
(A) The horizontal axis shows the number of nearest-neighbor IS pairs at a distance between 0.5 and 30 kb in the bacterial genomes examined. The p-value on the vertical axis is the likelihood that this number of IS pairs is greater than expected by chance alone, according to a randomization test described in Materials and Methods. There are two horizontal lines near the horizontal axis. The upper line represents a p-value of 0.01, the lower line a p-value of 0.00064, which is the Bonferroni-corrected value of p = 0.05, given that n = 78 independent statistical tests carried out for this analysis (0.00064 = 0.05/78).
(B) The randomized distribution of nearest-neighbor distance for IS91 in the genome of Rhodopirellula baltica. There are five members of this IS in the genome, and the closest pair of them is 3,051 bp apart. Based on this randomization test, this distance is significantly closer than expected by chance at a p = 0.0074.
(C) The horizontal axis shows the minimal distance between nearest-neighbor IS pairs that are between 0.5 and 30 kb apart in a genome. The vertical axis shows the likelihood that this minimal distance is observed by chance alone, based on the randomization test from (B) (described in Materials and Methods). The upper and lower horizontal lines represent Bonferroni-corrected p-values of p = 0.05 (0.00064 = 0.05/78) and p = 0.01 (0.000128 = 0.01/78). Both panels contain n = 78 datapoints derived from ISs in 28 different families.
doi:10.1371/journal.pcbi.0020162.g002
